# Ileal ureteral replacement for the management of ureteral avulsion during ureteroscopic lithotripsy: a case series

**DOI:** 10.1186/s12893-022-01690-0

**Published:** 2022-07-07

**Authors:** Changwei Yuan, Zhihua Li, Jie Wang, Peng Zhang, Chang Meng, Dan Li, Jingjing Gao, Hua Guan, Weijie Zhu, Boyu Lu, Zhichao Zhang, Ninghan Feng, Kunlin Yang, Xuesong Li, Liqun Zhou

**Affiliations:** 1 Department of Urology, Peking University First Hospital, National Urological Cancer Center, Institute of Urology, Peking University, No. 8 Xishiku St, Xicheng District, Beijing, 100034 China; 2 Department of Nursing, Peking University First Hospital, National Urological Cancer Center, Institute of Urology, Peking University, No. 8 Xishiku St, Xicheng District, Beijing, 100034 China; 3 Department of Urology, Emergency General Hospital, No. 29, Xibahenanli St, Chaoyang District, Beijing, 100028 China; 4 Department of Urology, Panjin Liaohe Oilfield Gem Flower Hospital, No. 26, YingBin St, Xinglongtai District, Panjin, 124010 China; 5 Department of Urology, Qinhuangdao Jungong Hospital, No. 15, YuFeng St, Haigang District, 066001 Qinhuangdao, China; 6 Department of Urology, Affiliated Wuxi No. 2 Hospital, Nanjing Medical University, No. 68, Zhongshan St, Liangxi District, Wuxi, 214001 China

**Keywords:** Ileal ureteral replacement, Ureteral avulsion, Ureteral injuries, Ureteroscopic lithotripsy, Laparoscopy, Robot-assisted surgery

## Abstract

**Introduction:**

To describe our initial experience with ileal ureteral replacement (IUR) for the management of ureteral avulsion (UA) during ureteroscopic lithotripsy.

**Methods:**

Between September 2010 and April 2021, ten patients received ileal ureteral replacement for ureteral avulsion during ureteroscopic lithotripsy. Anterograde urography and computed tomography urography (CTU) were applied to evaluate the lesion. Follow-up was performed with magnetic resonance urography and renal ultrasound as well as clinical assessment of symptoms. We retrospectively analysed the clinical data of ten patients treated with ileal ureteral replacement for the treatment of ureteral avulsion.

**Results:**

Four patients underwent open ileal ureteral replacement, two underwent laparoscopic ileal ureteral replacement, and four underwent robotic-assisted ileal ureteral replacement. The mean operative time (OT) was 310 min (range 191–530). The mean estimated blood loss (EBL) was 193 mL (range 10–1000). The mean length of the ileal graft was 21 cm (range 12–25). The median postoperative hospital time was 13 days (range 7–19). All surgeries were effectively completed, and no case required open conversion in laparoscopic and robotic-assisted surgeries. There was no obvious hydronephrosis according to contrast-enhanced computed tomography 3-dimensional reconstruction images without serious complications or progressive hydronephrosis during a median follow-up duration of 51 months (range 5–131), and the success rate was 100%.

**Conclusions:**

Our initial results and experience showed that ileal ureteral replacement for the management of ureteral avulsion during ureteroscopic lithotripsy is safe and feasible.

## Introduction

Urolithiasis is a common disease, so ureteroscopic lithotripsy is widely used in urology. With the prevalence and development of ureteroscopic surgery, treatment-related complications have recently increased. Ureteroscopy has been a common cause of iatrogenic ureteric trauma. The most severe complication is ureteral avulsion (UA), with an incidence of 0–0.3% [1]. UA, first introduced by Hodge to describe an upper urinary tract injury, refers to the discontinuation of the full thickness of the ureter [2].

There are some surgical reconstruction techniques for UA (longer ureteral injuries), including ileal ureteral substitution, autologous renal transplantation, and buccal mucosa ureteroplasty [3–5]. Autologous renal transplantation is not performed routinely by many medical centers, in addition to substantial trauma and nephrectomy-related organ loss. Although buccal mucosa ureteroplasty is another option for long segment ureteral injury, experience is limited [5]. Ileal ureteric replacement is a reliable solution for complex urinary reconstruction [6]. Open ileal ureteral substitution is a traditional surgical method, and it has gradually been replaced by minimally invasive surgery (MIS) in the clinic, including laparoscopic (LS) or robotic-assisted surgery (RAS) [7]. Currently, there is no consensus about the optimal surgical approach for the management of UA because of ureteroscopic lithotripsy.

In this study, we present the first and largest case series of ureteroscopy-related ureteral avulsion because of ureteroscopy treated with ileal ureter replacement.

## Materials and methods

### Clinical materials

Between September 2010 and April 2021, ten patients were admitted to the hospital with a diagnosis of UA treated with ileal ureter replacement, which was performed by the same surgeon. The UA of ten patients was caused by ureteroscopic lithotripsy in other hospitals. Two of the patients underwent immediate ureteroureterostomy and one underwent immediate IUR. Other seven patients underwent nephrostomy to wait follow-up operation. We retrospectively analyzed ten patients’ demographics, perioperative variables, and follow-up data, which are recorded in Table 1. The permission to use patient’s medical record and informed consent were obtained from the patients. This study was approved by the Ethics Committee of Peking University First Hospital, and informed consent was obtained from all patients.

**Table 1:** Summary of ten patients’ clinical materials

No.	Age	Gender	BMI (kg/m^2^)	From avulsion to IUR	Side of pathology	Surgical approach	Location of UA	The length of UA (cm)	The length of ileum (cm)	OT (min)	EBL (mL)	Liquid diet, days	Ambulation, days	Post-OPH, days	Post-OPC (C–D grade)	Follow-up (months)
1	52	Male	25.4	3 months	Left	Open	Lower	20	20	320	250	9	2	14	II (incomplete intestinal obstruction)	131
2	50	Female	22.3	12 months	Left	Open	Lower	22	22	360	100	7	3	12	None	124
3	46	Male	26.5	6 months	Left	Open	Upper	25	25	372	1000	6	3	13	None	92
4	61	Female	23.9	Immediate	Right	Open	Upper	17	25	530	100	6	3	19	None	74
5	36	Female	19.4	6 months	Left	LS	Lower	25	25	273	200	3	1	10	None	31
6	74	Male	21.3	6 months	Right	RA	Upper	12	12	238	20	4	1	11	None	16
7	39	Male	26.1	24 months	Left	RA	Middle	10	20	283	50	3	1	7	None	14
8	56	Female	18.9	5 months	Left	RA	Lower	20	20	191	10	5	1	11	None	10
9	36	Male	24.5	3 months	Right	LS	Upper	20	20	330	100	5	2	10	None	9
10	49	Female	27.9	6 months	Left	RA	Lower	20	20	201	100	3	1	18	II (incomplete intestinal obstruction)	5
Average, range	50 (36–74)	5 males; 5 females	23.6 (18.9–27.9)	–	3 right; 7 left	–		19 (10–25)	21 (12–25)	310 (191–530)	193 (10–1000)	5 (3–9)	2 (1–3)	13 (7–19)	–	51 (5–131)

*BMI* body mass index, *C–D grade* Clavien–Dindo grade, *EBL* estimated blood loss, *IUR* ileal ureteral replacement, *LS* laparoscopy, *OPC* operative complications, *OPH* operative hospitalization, *OT* operative time, *RA* robotic-assisted, *UA* ureteral avulsion

### Presurgical evaluation

**Fig. 1:**
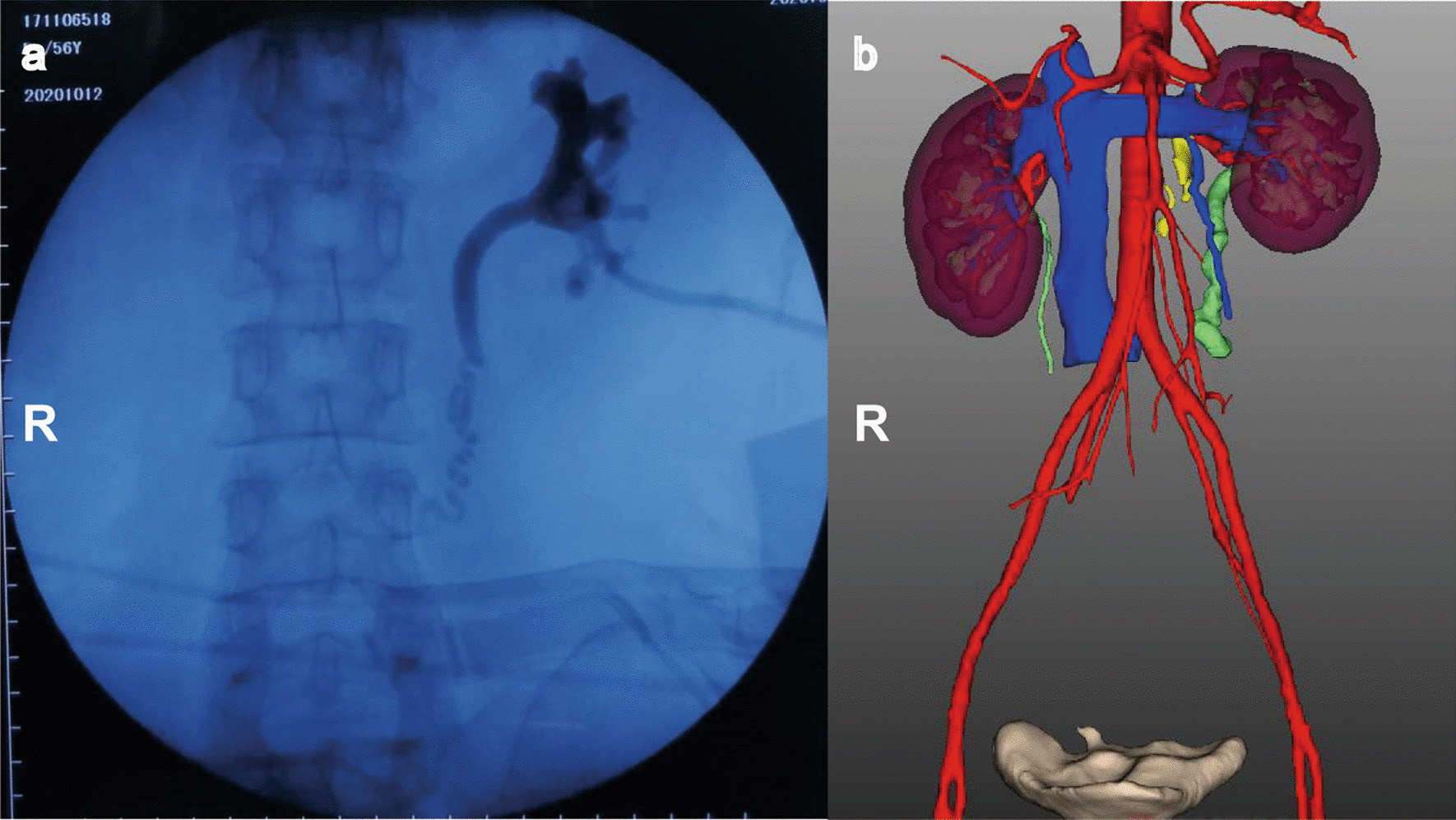
Perioperative examination was performed to evaluate ureteral avulsion. **a** Antegrade pyelography from the nephrotomy tube demonstrating that ureteral contrast medium was interrupted with mild hydronephrosis. **b** Preoperative three-dimensional image reconstruction demonstrates the location of UA

Preoperative radiographic examinations, such as antegrade pyelography and computed tomography urography (CTU), were performed routinely. These patients were revealed by preoperative antegrade pyelography by nephrostomy tube (except case 4) (Fig. 1). Antegrade pyelography demonstrated that the ureteral contrast medium was interrupted with or without hydronephrosis. Preoperative three-dimensional (3D) image reconstruction was applied to evaluate the relationship between the ureter and adjacent organs (Fig. 1). Renal dynamic imaging was used to evaluate perioperative renal function. Renal function and serum electrolytes were normal in all patients. Open surgery was the main method before the laparoscopic and robotic technique was applied in the clinic. When explaining the difference between the two approaches, the robotic-assisted or conventional laparoscopic approach depends on the patient’s choice.

#### Surgical techniques

**Fig. 2:**
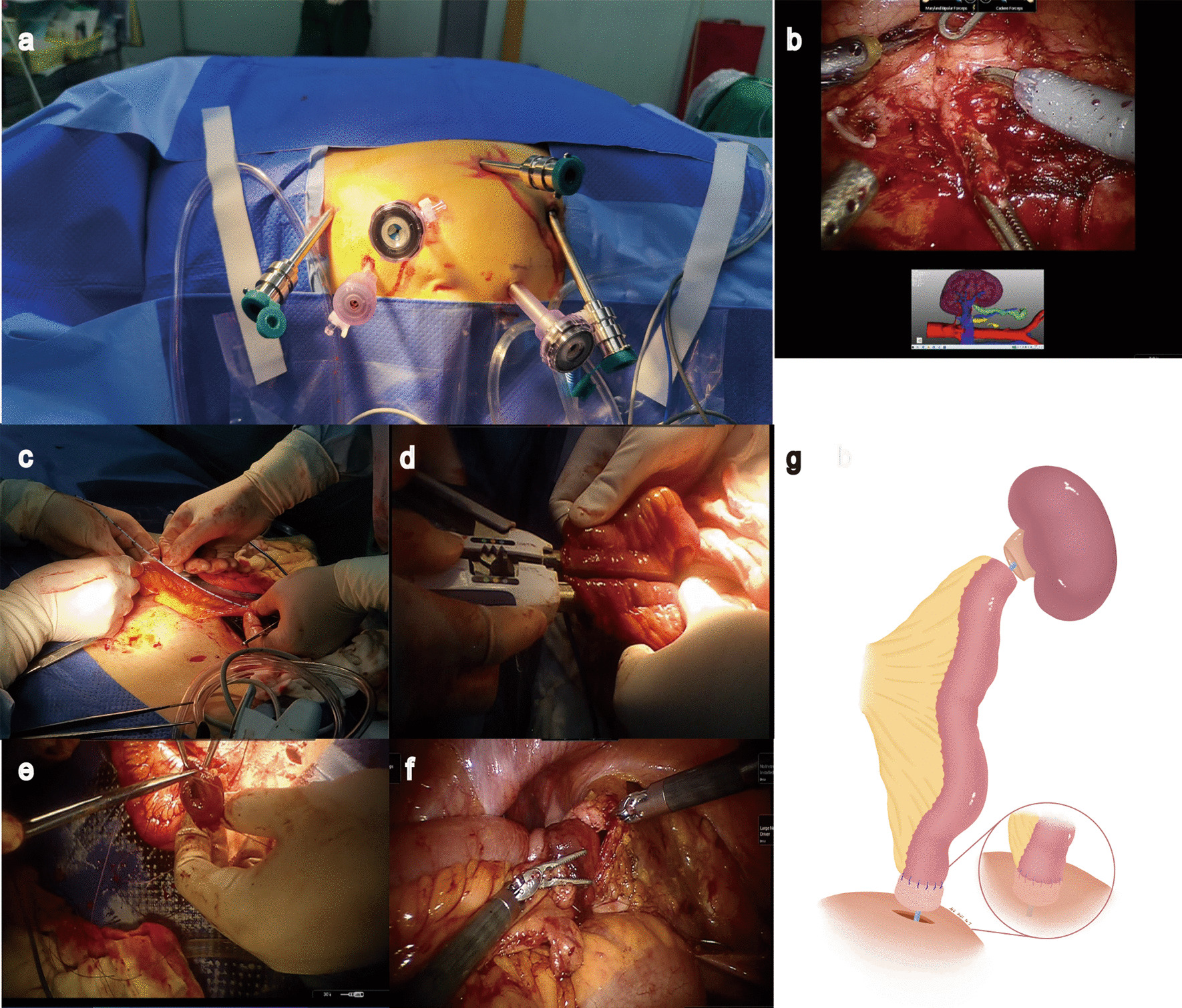
The pictures of operation. **a** Port placement of minimal invasive IUR. **b** Intraoperative navigation by 3D models in robotic surgery; **c** the length of the ileal segment was measured; **d** the side-to-side anastomosis was created at the edge of the anti-mesentery by a linear stapler; **e** the anti-reflux nipple valve was made; **f** ileovesical anastomosis; **g** schematic diagram of the anti-reflux nipple valve

The ileal ureter substitution was similar to that described in our previous study [7]. After general anesthesia, the patient was placed in the oblique position (45° from horizontal) with the lesion side up. Access was achieved using a Veress needle, and abdominal ports were placed (Fig. 2a). The supine position was obtained with an abdominal midline incision of approximately 25 cm in open surgery. The surgical procedures were similar in laparoscopic and robotic ileal ureteral replacement. After mobilization of the colon, the renal pedicle was located through the gonadal veins and ureter. The ureter was dissected free to the level of the lesion, which was recognizable because of obvious scar tissue (Fig. 2b). The ureter adjacent to the lesion was widely spatulated for the anastomosis. The ileal segment was selected 12–20 cm away from the ileocecal junction after measuring the length of the defect (Fig. 2c), which was used to bridge the ureteral defect in an isoperistaltic way. The ileal graft was exteriorized through a midline infra-umbilical incision. Intestinal continuity was restored after the ileal segment was resected with side-to-side anastomosis by linear staplers (Fig. 2d). A mesenteric window is created, and the ileal segment is played through into the retroperitoneal space while the distal part is close to the bladder. A 7F ureteral stent was inserted and fixed to the proximal and distal ends of an ileal graft to avoid dislocation. Subsequently, the ileal graft was returned to the abdominal cavity, and the pneumoperitoneum was reestablished. Pyeloileal and ureteroileal anastomoses were performed in an intermittent end-to-end fashion with the 4-0 vicryl. A distal anti-reflux nipplevalve was created (Fig. 2e). The anterior wall of the bladder was cut, and ileal‐vesical anastomoses were performed using full‐thickness intermittent sutures (Fig. 2f, g). Then, the colon was placed back in place and covered in front of the ileal graft. Close the incised mesentery to prevent internal hernias. Finally, a Foley catheter was left to the bladder, and two drains were placed adjacent to the anastomoses.

##### Postoperative care and follow-up

After the operation, patients received an indwelling Foley catheter, a drain near the anastomosis, and a Double-J stent (D-J stent). The Foley catheters were removed within 1 week postoperatively. Radiography was performed routinely to confirm that the D-J stent stayed in the appropriate position. The D-J stent was removed 2 or 3  months after surgery. After that, antegrade pyelography was performed to judge whether the upper urinary tract was unobstructed before the nephrostomy tube was removed. Patients were followed up for 3 months, 6 months for the first year, and annually after surgery by a standardized and telephone interview or clinical visit. Physical examination, blood tests (including arterial blood gas analysis) and routine urine tests were performed routinely. Renal function was assessed by serum creatinine every visit and diuretic renal dynamic imaging at half and 1 year. About the follow-up plan of radiographic examinations, magnetic resonance urography (MRU) was performed at the 3rd month, CTU at the 6th month. Ultrasound was performed every 3  months in first year and semiannual evaluation later. The success of surgery was defined as improved or no progress in hydronephrosis on ultrasound without nephrostomy tube.

## Results

**Fig. 3:**
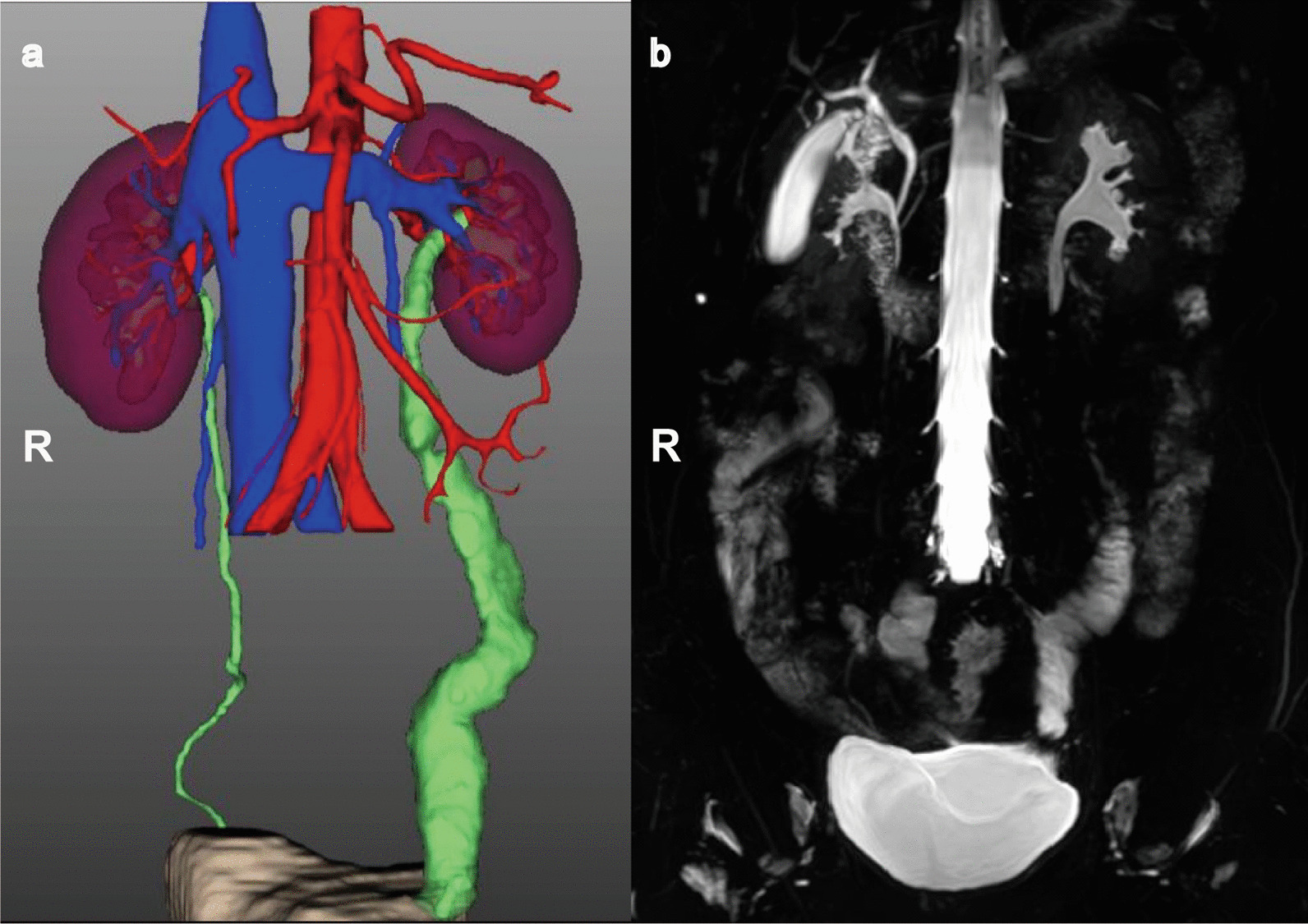
Postoperative evaluation at the follow-up. 3D CT image reconstruction (**a**) and magnetic resonance urography (**b**) demonstrate the morphology of ileal ureter replacement without hydronephrosis

All patients were diagnosed with UA caused by ureteroscopic lithotripsy with the nephrostomy tube performed intraoperatively or postoperatively for 3 to 6 months (except case 4) before IUR. Patient 5 and 7 previously underwent immediate ureteroureterostomy prior to IUR. The result in detail is described in Table 1. The mean age of the patients was 50 years (range 36–74), and the mean body mass index (BMI) was 23.6 kg/m^2^ (range 18.9–27.9). Four patients underwent open IUR, two underwent laparoscopic IUR, and four underwent robotic-assisted IUR. The mean operative time (OT) was 310 min (range 191–530). The 530 min of OT (only patient 4), is of the whole procedure from the beginning of ureteroscopic lithotripsy to the end of IUR. The mean estimated blood loss (EBL) was 193 mL (range 10–1000). The mean length of the ileal graft was 21 cm (range 12–25). All surgeries were effectively completed, and no case required open conversion in laparoscopic and robotic-assisted surgeries. No postoperative complications of high grade (grade III and IV) occurred within 1 month of surgery according to the Clavien–Dindo classification system [8]. Two patients had incomplete intestinal obstruction (grade II) and were treated with short-term fasting water (case 1 and case 10). Except that, no other complications occurred in all patients. The mean liquid diet time was 5 (3–9) days, and the ambulation time was 2 (range 1–3) days. The median postoperative hospital time was 13 days (range 7–19). Postoperative renal function, shown by serum creatinine and diuretic renal dynamic imaging, were in the normal range in ten patients. To date, the median follow-up duration has been 51 months (range 5–131). All patients had nephrostomy tubes, and D-J stents were removed 2–3 months after the operation. There was no obvious hydronephrosis according to the contrast-enhanced CT 3D reconstruction image (Fig. 3a). MRU showed well-healed anastomosis, and the upper urinary tract was unobstructed (Fig. 3b). Therefore, there was a 100% success rate without serious complications or progressive hydronephrosis during follow-up at 5 to 131 months. All patients were in good general condition and did not report obvious discomfort during follow-up.

## Discussion

As ureteroscopic technology has advanced, as minimally invasive surgery, ureteroscopic lithotripsy intervention has become an increasingly common treatment for patients with renal or ureteral stones. Most of the complications caused by this technology and management respond favorably to simple drainage of urine with D-J stents or ureteral stents. However, iatrogenic ureteral avulsion is a disastrous complication that can occur during a ureteroscopic procedure, although such a complication is a rare occurrence [9].

Ureteral avulsion might occur for the following reasons: (1) ureter inflammatory edema or hyperplasia scar formation and other changes, resulting in distortion and thinning of the ureter. (2) If a rigid ureteroscope that is too large is placed into the ureter, the phenomenon of scabbling of the ureteroscope easily occurs when it is withdrawn. (3) The proximal third of the ureter may be at greatest risk for avulsion because of the least muscular tissue support and low tensile strength [10]. There are several methods to avoid ureteral avulsion. For example, strictly grasping operative indications, especially the size and location of stones, should be considered. Then, it is essential for safety to place a working guidewire when performing ureteroscopic lithotripsy [11]. Moreover, maintaining a good view and gentle operation consistently avoids rough operation.

When UA occurs, some reasoned approaches for the treatment of UA should be adopted. Although it may be tempting to perform immediate repair once injured, the surgeon’s experience and center facilities must be taken into account. One patient (case 4) with ureteral avulsion underwent immediate laparotomy and ileal ureter replacement by a skilled doctor. If not, in general, delayed repair is recommended, and diversion of the urine (e.g., nephrostomy) will be conducted. If conservative management is attempted, there is a high risk of renal failure and stricture, even nephrectomy, whenever reconstruction is performed [12, 13]. There is no consensus over how to reconstruct the damaged ureter. The location and length of the ureteral defect were evaluated by preoperative radiology, and the reconstruction strategy depended on it.

Autologous renal transplantation is described as a choice for ureteral injury, but it is not performed routinely by many medical centers, in addition to substantial trauma and nephrectomy-related organ loss. Therefore, this operation has been decreasing continuously in recent years [14]. Some studies have shown that appendiceal interposition can be effective for UA [15, 16]. This is generally suitable for middle and distal ureteral defects because of the anatomical location of the appendix. In addition, Duty et al. [17]. reported that six patients had a good outcome after undergoing appendiceal onlay flap ureteroplasty for the proximal and middle ureters, but the mean stricture length was only 2.5 cm. Therefore, this technique may be more suitable for short defects. In addition, buccal mucosa ureteroplasty is another option for long segment ureteral injury, but experience is limited [5]. In line with appendiceal interposition, this technique for reconstruction defect length was approximately 3–5 cm [18], which was difficult for long UAs. Furthermore, bladder flap with psoas hitch is an optional way to long segment injuries (mostly no more than 10 cm) [19], overlong lesion will lead to excessive tension at the anastomosis. UA reached a length of 10–25 cm in our study, and it was difficult to dissect due to extensive fibrosis around the ureter and the loss of normal tissue anatomy, so that was not suitable for our patients. By the way, our previous study also found IUR combined Boari flap–psoas hitch could be applied to these patients with borderline renal function in order to minimize the length of ileal segment [20].

A longer ureteral defect can be replaced using a segment of the intestines, usually the ileum (ileal interposition graft), in our department [7]. The first ileal ureter substitution was reported by Goodwin et al. [21]. This technique is regarded as an effective procedure for repairing long ureteral defects [22].

IUR may also be the first choice for ureteral avulsion regardless of immediate or delayed repair, and ureteroureterostomy was not a recommended attempt. An avulsed ureter with severe inflammation would cause ureteral stricture if immediate ureteroureterostomy was performed. These conditions occurred in cases 5 and 7, and the two patients had hydronephrosis soon after immediate ureteroureterostomy. Finally, IUR was performed, and hydronephrosis was released during follow-up. Open ileal ureteral substitution has some disadvantages, including greater trauma, longer recovery, and more complications. At present, minimally invasive techniques are often chosen, including laparoscopic surgery and robotic surgery [7, 23], which have lower narcotic requirements, shorter hospital stays, and shorter times to convalescence. According to Table 1, mean operative time as shorter for MIS group. Results concerning blood loss, liquid diet, and ambulation time proved that minimally invasive techniques might conform more to the principle of enhanced recovery after surgery (ERAS). However, incomplete intestinal obstruction (cases 1 and 10) might occur regardless of the surgical approach chosen.

Nephrostomy is performed routinely to ensure normal preoperative renal function. Patients with significant renal insufficiency should not be recommended for surgery. There is still no standard length of time to keep the nephrostomy tube, and the length of time less influences the prognosis based on our initial experiences. The operation should conform to some principles to ensure successful anastomosis. It is widely acknowledged that the general principles of ureteral reconstruction include a good remaining blood supply, a tension-free, watertight anastomosis that is adequately spatulated, and the use of absorbable fine sutures [24, 25].

There is still controversy regarding whether an anti-reflux papillary valve is recommended. Xu et al. [26] reported that the proximal anti-refluxing technique appears to be a reliable procedure for treating long-segment ureteral strictures. Waldnerd et al. [27]. thought that anti-reflux procedures were not always necessary because ileal peristalsis could suppress the reflux, especially more than 15 cm of ileum. According to our previous studies [7, 20], proximal ureteral ileum anastomosis should not be designed to prevent reflux, but proximal anastomosis should be as wide as possible to allow urine to flow out without resistance, while distal anastomosis is necessary to prevent reflux. In this cohort, UA reached a length of 10–25 cm, so IUR may be the optimal method to bridge the defect, which makes patients eliminate nephrostomy and resume a normal life. We used a distal anti-reflux nipple for the anti-flux procedure. No cases of postoperative reflux were observed by cine MRU.

There are some postoperative complications, including urinary infection, metabolic acidosis, mucus obstruction, or stenosis of the ileal ureter. Therefore, postoperative management of the patients was also necessary. Two patients had incomplete intestinal obstruction (grade II) and were treated with short-term fasting water in our study.

The scarcity of literature, as well as small case series, reflects the exploration of IUR for UA [12, 13, 28]. Our study reported that ten patients had the longest follow-up range of 5 to 131 months, demonstrating the treatment and consideration of ureteral avulsion under ureteroscopy, which is rare. In addition, preoperative three-dimensional reconstruction was first used in the management of UA to help assess the anatomic relationship between the targeted area and peripheral structure and improve surgical efficacy, which is not reported in the UA literature. Our experience with IUR, whatever MIS or open surgery, shows the feasibility in the treatment of UA because of ureteroscopy.

There are some limitations to our study. It is a retrospective study and has some bias. Our results are limited by the small number of patients. It should be mentioned that UA is relatively rare, so large series are difficult to generate. An important drawback is the lack of control group that would enable to compare success rate and complications rates. We are optimistic that this initial report can serve as a foundation for developing standardized management of UA.

## Conclusions

In conclusion, our initial experience demonstrates that ileal ureteral replacement is a feasible and effective technique for managing ureteral avulsion because of ureteroscopic lithotripsy. However, future studies including large numbers and long-term follow-up of cases are required.

## Data Availability

The datasets generated and/or analyzed during the current study are not publicly available due to privacy or ethical restrictions but are available from the corresponding author on reasonable request.
